# An assessment of the role of socio-economic, maternal and service utilization factors in increasing self-reported maternal complications in India

**DOI:** 10.1186/s12884-021-03997-x

**Published:** 2021-07-21

**Authors:** Pradeep Kumar, Shobhit Srivastava, Chanda Maurya, Preeti Dhillon

**Affiliations:** Department of Mathematical Demography and Statistics, International Institute for Population Sciences, Mumbai, Maharashtra India

**Keywords:** Maternal complications, Decomposition; NFHS, India

## Abstract

**Background:**

Self-reported maternal complications are associated with maternal morbidity, deliveries by C-section, postpartum depression, and maternal death. Thus, it is necessary to examine the contribution of socio-demographic and maternal characteristics, as well as service utilization in the rising self-reporting of maternal complications (difficulty with daylight vision, convulsions, swelling of the legs, body or face, heavy vaginal bleeding or high fever) in India. The study aimed at examining the factors that have influenced the increasing prevalence of maternal complications between 2005–06 and 2015–16 in India.

**Methods:**

Data from the two most recent rounds of the National Family Health Survey, which covered a sample of 36,850 and 190,898 women respectively who delivered in the last five years preceding the survey has been used. Logistic regression analysis was performed to carve out the factors which significantly contributed to maternal complications among women aged 15 – 49 years in India. With the help of the Fairlie decomposition technique, the study quantified the contribution of factors which influenced the changes in maternal complications in the period from 2005–06 to 2015–16.

**Results:**

A significant increase was seen in the prevalence of maternal complications — from 43.6% to 53.7% between the years 2005–06 and 2015–16. About 21% of the increase could be explained by certain maternal, households level factors, service utilization and birth outcomes. For example, service utilization, in which 13% was attributed to the place of delivery and 6% to postnatal care, was the major contributor to the increase in maternal complications from 2005–06 to 2015–16). This was followed by individual-level factors like education (2%), body mass index (4%) and tobacco use,. It was also found that household-level factors like standard of living (-3.7%) and region (-1.4%), and birth weight contributed to the reduction of complications during the period.

**Conclusion:**

The increase in the prevalence of maternal complications in India could be attributed mainly attributed to increase in reporting behavior, an outcome of increased utilization of maternal healthcare services, and increase in BMI. However, reduced prevalence of maternal complications can be attributed to the decrease in the prevalence of low-birth-weight babies and tobacco use among women in India.

**Supplementary Information:**

The online version contains supplementary material available at 10.1186/s12884-021-03997-x.

## Background

Maternal complications have adverse effect on maternal health and hence should be identified targeted for treatment [1, 2]. A maternal complication is defined as “any health condition attributed to complicating pregnancy and childbirth, and which harms a woman’s well-being and functioning” [3]. Maternal complications during pregnancy, delivery, and after birth are responsible for a significant number of preventable deaths among women. Every year, about 350,000 maternal deaths occur globally due to maternal complications, most of which are take place in developing countries [4]. According to the World health organization (WHO), globally, there was a decrease in maternal mortality — from 451,000 to 295,000 — from the years 2000 to 2017 [5]**.** In India, pregnancy- and childbirth-related deaths dropped significantly from 103,000 in 2000 to 35,000 in 2017, a 55% decrease in the numbers [6]. These improvements are earlier assessed in relation to the progress of Government initiatives towards achieving maternal healthcare goals [6].

Several individual-level risk factors are associated with the maternal complications, such as maternal age, presence of co-morbidities or medical conditions, multiple pregnancies, and previous miscarriage [7, 8]. Behavioural factors like alcohol and tobacco consumption increase the risk of maternal complications like preterm childbirth, low birth weight, postpartum hemorrhage, placenta previa, abruptic placentae, congenital anomalies, and sudden infant death syndrome [9–12]. Furthermore, nutrition factors like Body Mass Index of women, and anaemia among them, also are significantly associated with maternal complications, including gestational hypertension, gestational diabetes, preeclampsia, and pregnancy terminated by caesarean section [13], as well as and poor pregnancy outcomes [14].

Social factors like wealth index, place of residence, availability of medical services, and distance from medical facilities were significantly related to maternal complications. According to Sontakke et al. (2009), obstetric morbidity was higher among women residing in rural areas and with a low standard of living [15]. Further, utilization of maternal healthcare services like several antenatal visits and institutional deliveries [16], and birth outcomes like multi-parity [14] and twin pregnancy [17] are also significant factors in maternal complications. Women who suffer from any sort of maternal complications during the postpartum period are more likely to seek assistance from skilled health providers [18] and stay longer at the healthcare facility after childbirth [19].

**Fig. 1:**
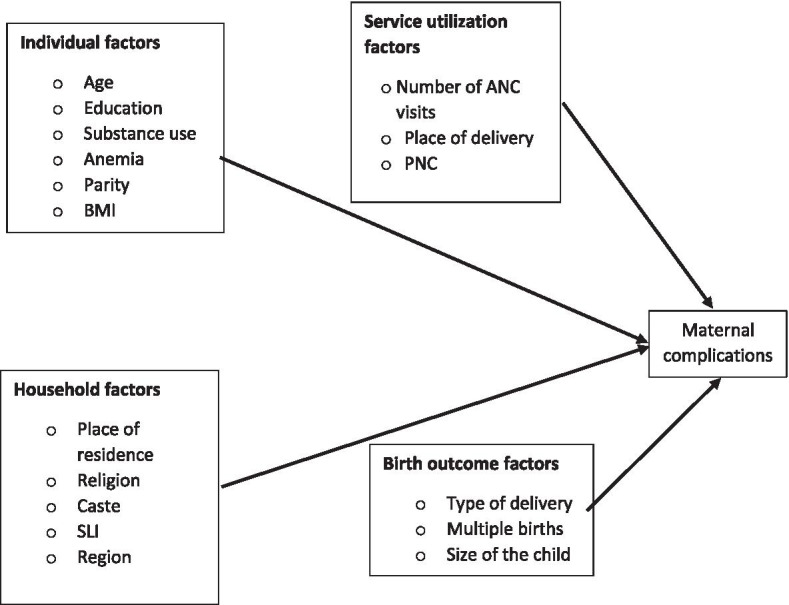
Conceptual Framework

Earlier studies of maternal complications showed only their determinants or the factors associated with them [15, 20–22]. There is a dearth of research that is focused on the changes in maternal complications and their contributing factors in India. Hence the present study aims to identify the determinants of maternal complications (difficulty with daylight vision, convulsions, swelling of the legs, body or face, heavy vaginal bleeding or high fever) among women aged 15 – 49 years. in India. With the increase in maternal complications, and changing socio-demographic and maternal factors, it is essential to examine the contribution of these factors in the increased self-reporting of maternal complications during the last decade (2005–06 to 2015–16). Thus, the analysis in this study can provide useful insights on whether the increasing prevalence of maternal complications was due to the changing demographics and health condition of Indian women, or indicative of a rising trend in self-reporting of complications. Therefore, the aim of this study is to describe the increasing maternal complications in India, as well as to quantify the contribution of selected maternal factors, household factors, service utilization and birth outcomes factors to the rising trend of self-reporting of maternal complications by using decomposition methods. The conceptual framework for the study of the association between these factors and the outcome variable (maternal complication) is shown in Fig. 1.

## Methods

Data used for analysis in this study were collected in two recent rounds of the National Family Health Survey (NFHS), NFHS 3 and NFHS 4, which were conducted, respectively, in 2005–06 06 and 2015–16. The NFHS series is a nationally representative cross-sectional survey which is conducted under the stewardship of the Ministry of Health and Family Welfare (MoHFW) of India. NFHS collects information on population, health and nutrition status, abortion, sexual behavior, knowledge of HIV/AIDS, and domestic violence from all states and union territory [23, 24]. The survey used a stratified two-stage sampling procedure to draw samples that are representative of the population. In the third and fourth rounds, standard questionnaires were administered to the respondents after obtaining their written consent to participate in the survey. A total of 124,385 women in NFHS 3 and 699,686 women in NFHS 4 were interviewed with a response rate 94.5% and 97% in the respective surveys. The effective sample size was 36,850 women for NFHS-3 and 190,797 women for NFHS-4. Only those women, who had at least one birth in the last five years preceding the survey, were included in the sample.

### Outcome variable

Large-scale surveys seek limited information on complications during pregnancy, delivery, and post-delivery [1, 22–24]. Based on the information available from past research, we defined the outcome variable to be women who had any maternal complication in their last pregnancy, delivery, and/ or post-delivery in the five years preceding the survey. Data on maternal complications were obtained from self-reported pregnancy and post-delivery complications. The women were asked —with response options of ‘yes’ or ‘no’ — if they had experienced difficulty with daylight vision (1 “Yes,” and 0 “No”), convulsions not related to fever (1 “Yes,” and 0 “No”), swelling of the legs, body or face (1 “Yes,” and 0 “No”), heavy vaginal bleeding (1 “Yes,” and 0 “No”), or high fever (1 “Yes,” and 0 “No”). These variables, which were common in both rounds of the NFHS, were identified as the outcome variable on maternal complications — a value of 1 if they were present or 0 for their absence — as reported by the women.

### Predictor variables

Individual-level predictor variables included maternal age (15 – 19, 20 – 24, 25 – 29, 30 –34 and 35 + years), highest maternal highest education (no education, primary, secondary and higher), substance (alcohol or tobacco) use (with a response option of yes or no) anemic (no or yes), parity (one, two, three and four & above), and the Body Mass Index (BMI) (Underweight, Normal and Obese).

Household-level variables included place of residence (Urban or Rural), religion (Hindu, Muslim, and Others), caste (Scheduled Caste (SC) or Scheduled Tribe (ST) and non-SC or ST which included Other Backward Class (OBC), and other caste groups), and standard of living index, SLI, which is classified as low, medium, and high as household level variables.

The regions were categorized into North, Central, East, North-East, West, and South. Information on these predictor variables was directly obtained in the survey. The Standard of Living Index (SLI) is taken as a proxy measure of household income. NFHS provides a wealth index as a proxy measure for income, which is based on responses to questions related to the household amenities and conditions, which is not comparable with SLI. Moreover, the value of the assets owned by the respondents are likely change over time because of inflation and other factors. To overcome the above shortcomings, we constructed the Standard of Living Indices for both rounds of the survey, the values of which are comparable. The SLI scores were used to categorize the respondents into three categories — low, medium, and high — using three-percentile distribution. Categorization used the same percentile cut-offs for NFHS-3 and NFHS-4.

Service-level factors included number of antenatal visits made by the respondents during their pregnancy (no visits, 1–3 visits, and 4 and more visits), place of delivery (home or institutional, whether public or Private), and post-natal care (PNC) received (no PNC, PNC received more than 48 h after, and within 48 h). These were considered as explanatory variables for the pregnancy complications of the respondents. Finally, birth outcomes included multiple births (single or twins), size of the child (large, average or small), mode of delivery (normal or C-section).

### Statistical analysis

Descriptive analysis was used to determine the differences in maternal complications in 2005–06 and 2015–6 according to background variables. For comparing the prevalence of complications (significant change) in NFHS-3 and NFHS-4, the study used a test of proportion for the two samples.

The test statistic for comparing two proportions is defined as:

$$Z=\frac{{\widehat{p}}_{1}-{\widehat{p}}_{2}}{\sqrt{p\dot{*}\left(1-p\dot{*}\right)\left(\frac{1}{{n}_{1}}+\frac{1}{{n}_{2}}\right)}}\sim N\left(\mathrm{0,1}\right)$$

where $p\dot{*}=\frac{{n}_{1}{\widehat{p}}_{1}+{n}_{2}{\widehat{p}}_{2}}{{n}_{1}+{n}_{2}}$; ${\widehat{p}}_{1}$ and ${\widehat{p}}_{2}$ are respectively the proportions of maternal complication use in the two periods (2005–06 and 2015–16); and ${n}_{1}$ and ${n}_{2}$ are the respective sample sizes in the two rounds of surveys.

Logistic regression was used to carve out the significant factors that contributed to maternal complications among women aged 15 – 49 years in India [25]. To identify the underlying causes of the difference in the prevalence of maternal complications between the two surveys, a decomposition technique was used which is now a common employed approach for identifying and quantifying inter-group differences.

For computing the differences between the two sample groups (of 2005–06 and 2015–16) in the prevalence of maternal complications, and to decompose these differences into their major contributing factors, Fairlie’s decomposition method was used [26]. The use of decomposition methods is widely attributed to Blinder (1973) and Oaxaca (1973). Their technique, however, is not appropriate if the outcome variable is dichotomous, such as maternal complications, which is coded as 0 for a “no” and 1 for a “yes”. Hence, we used the extension of the Blinder-Oaxaca technique, which is Fairlie decomposition, which is appropriate for binary models, for decomposing the decadal change in the prevalence of maternal complications to identify the contributions made by individual factors to the overall prevalence [26].

$${Y}^{t1}-{Y}^{t2}=\left[\sum_{i=1}^{{N}^{t1}}\frac{F\left({X}_{i}^{t1}{\beta }^{t2}\right)}{{N}^{t1}}-\sum_{i=1}^{{N}^{t2}}\frac{F\left({X}_{i}^{t2}{\beta }^{t2}\right)}{{N}^{t2}}\right]+\left[\sum_{i=1}^{{N}^{t1}}\frac{F\left({X}_{i}^{t1}{\beta }^{t1}\right)}{{N}^{t1}}-\sum_{i=1}^{{N}^{t1}}\frac{F\left({X}_{i}^{t1}{\beta }^{t2}\right)}{{N}^{t1}}\right]$$

Here, Y is the dependent variable (maternal complications) at time t_1_ (2005—06) and t2 (2015–16), ${N}^{J}$ is the sample size for time t, ${X}^{J}$ is the row vector of average values of the independent variable and ${\beta }^{J}$ is the vector of coefficient estimates for time t. This method of decomposition allows us to quantify the absolute contribution of factors explaining the decadal change (2005–06 to 2015–16) in the probability of having maternal complications among women in India. STATA 14 was used to carry out the analysis [27].

## Results

A higher proportion of women belonged to the 20–29 years age group in both rounds of the survey. The proportion of educated women increased from 62 to 71% between 2005–06 and 2015–16. Interestingly, substance use among women had decreased during this period, as also the prevalence of anemia (from 57 to 55%). In the same period, the proportion of underweight women had also decreased, and the percentage of small size babies decline from 22 to 14% (Additional file 1).

**Fig. 2:**
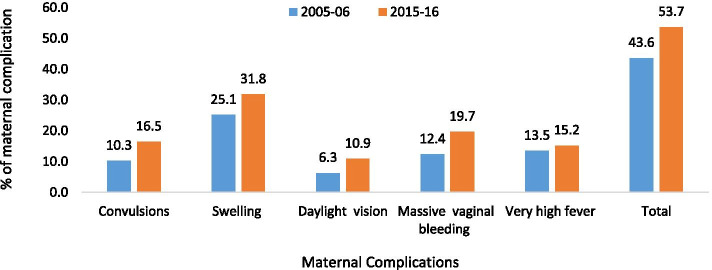
Changes in reporting of different maternal complications in India between 2005–06 and 2015–16

Figure 2 represents the change in types of maternal complications in India from 2005–06 to 2015–16. It was found that there was an increase in self-reporting of each type of maternal complication, which leads to about a 10% increase in the overall prevalence of maternal complication in the decade. The highest increase was in the reporting of massive vaginal bleeding (7%), followed by convulsions and swelling (6% each), and reduced daylight vision (5%).

The percentage of women who reported any pregnancy complication during the last birth in the last five years preceding the survey by their background characteristics is presented in Table 1. Overall, there was a significant increase in the proportion of maternal complications during the decade between the two surveys (from 43.6% to 53.7%; *p* < 0.001). This change is observed across all factors — individual, household, service utilization, and birth outcomes.

**Table 1:** Percentage of women who reported any pregnancy complication during last birth in past five year preceding the survey by selected background characteristics in India, (NFHS3, 2005–06 & NFHS4, 2015–16)

Variables	NFHS-3 (2005–06)			NFHS-4 (2015–16)			*p*-value for change
	***N* = 36,850**	**95% CI**		***N* = 190,898**	**95% CI**		
Age (years)							
15–19	45.7	43.6	47.8	53.9	52.6	55.1	0.001
20–24	43.5	42.6	44.5	53.7	53.3	54.2	0.001
25–29	43.0	42.2	43.9	53.3	52.9	53.7	0.001
30–34	43.5	42.3	44.6	54.5	54.0	55.1	0.001
35 and Above	44.6	43.1	46.1	53.1	52.5	53.8	0.001
Education							
No education	44.6	43.8	45.4	53.5	53.1	53.9	0.001
Primary	44.7	43.4	46.1	53.6	53.0	54.2	0.001
Secondary	41.6	40.8	42.4	52.9	52.6	53.2	0.001
Higher	43.3	41.6	44.9	56.8	56.1	57.5	0.001
Substance use							
No	43.1	42.6	43.7	53.6	53.4	53.9	0.001
Yes	47.7	46.4	49.1	54.3	53.6	55.0	0.003
Anaemia status							
No	43.0	42.2	43.9	53.8	53.4	54.1	0.001
Yes	44.9	44.2	45.6	53.7	53.4	54.0	0.001
Parity							
One	46.1	45.2	47.1	56.2	55.8	56.5	0.001
Two	40.6	39.7	41.6	51.6	51.2	52.0	0.001
Three	42.1	40.9	43.3	52.3	51.7	52.8	0.001
Four and above	45.3	44.3	46.3	54.4	53.8	54.9	0.001
BMI status							
Underweight	43.0	42.1	44.0	51.8	51.3	52.3	0.001
Normal	44.6	43.9	45.4	53.3	53.0	53.6	0.001
Obese	44.5	42.8	46.2	56.9	56.3	57.5	0.001
Residence							
Urban	44.6	40.2	41.8	53.8	52.9	53.8	0.001
Rural	41.0	43.9	45.2	53.4	53.5	54.1	0.001
Religion							
Hindu	42.4	41.8	43.0	53.4	53.1	53.6	0.001
Muslim	48.5	47.2	49.8	54.0	53.5	54.6	0.001
Others	46.5	45.1	47.8	57.2	56.5	57.8	0.001
Caste							
SC/ST	42.8	41.96	43.7	53.5	53.2	53.9	0.001
Non SC/ST	44.0	43.33	44.6	53.7	53.4	54.0	0.001
SLI							
Low	45.8	44.9	46.7	54.8	54.3	55.2	0.001
Medium	41.8	41.0	42.5	52.7	52.4	53.0	0.001
High	42.7	41.4	43.8	55.7	55.1	56.2	0.001
Region							
North	41.4	40.2	42.6	53.8	53.3	54.3	0.001
Central	44.0	42.9	45.1	58.7	58.3	59.1	0.001
East	52.1	50.8	53.4	53.3	52.8	53.8	0.001
Northeast	46.6	45.4	47.7	41.1	40.6	41.7	0.001
West	37.7	36.2	39.2	47.4	46.5	48.2	0.001
South	35.8	34.6	37.1	54.3	53.6	54.9	0.001
Number of ANC visits							
No visit	44.3	45.3	47.6	52.0	52.4	53.4	0.001
1–3 visits	43.4	42.5	44.2	53.6	53.5	54.3	0.001
4 & more visits	43.7	41.4	42.9	54.7	53.5	54.1	0.001
Place of delivery							
Home	44.2	43.5	44.9	52.1	51.6	52.6	0.001
Institutional	42.8	42.1	43.5	54.0	53.8	54.3	0.001
PNC							
No or more	44.6	43.9	45.3	51.8	51.4	52.2	0.001
Within 48 h	42.0	41.2	42.7	54.7	54.4	54.9	0.001
Mode of delivery							
Normal	43.4	42.8	43.9	52.8	52.6	53.1	0.001
C-Section	46.0	44.5	47.4	57.2	56.6	57.8	0.001
Multiple births							
Single	43.5	43.0	44.1	53.6	53.4	53.8	0.001
Twin	52.6	47.1	58.1	62.9	60.6	65.1	0.035
Size of child							
Large	43.6	42.6	44.7	54.5	54.0	55.2	0.001
Average	41.8	41.1	42.5	52.7	52.5	53.0	0.001
Small	47.9	46.9	49.0	57.3	56.7	57.9	0.001
Total	**43.6**			**53.7**			0.001

*Note*: *CI* Confidence Interval

*NFHS* National Family Health Survey, *BMI* Body Mass Index, *SLI* Standard of Living Index, *ANC* Anti-Natal Care, *SC/ST* Scheduled caste/Scheduled tribe, *PNC* Post-Natal Care, *C-Section* Caesarean Section

### Individual factors

In 2005–06, the proportion of self-reported complications was most (45.7%) among mothers aged 15 – 19 years than in the other age groups, whereas, in 2015–16, it was highest among women aged 30 – 34 years (54.5%). Women who used substances (tobacco or alcohol) reported a higher proportion of complications in both the survey periods (47.7% in 2005–06 and 54.3% in 2015–16). In 2005–06, a higher proportion of anemic women reported complications (44.9%) than those who were not. Further, in 2005–06 and 2015–16 women with first parity reported more complications (46.1% and 56.2% respectively) than did women with higher parities. The percentage of maternal complications among obese women was higher (44.7% and 56.9% in 2005–06 and 2015–16 respectively) than in the other BMI groups.

### Household factors

A higher percentage of urban women reported maternal complications in both survey rounds (44.6% and 53.8% respectively) as compared to women from rural areas. One observation of interest is that high percentages of maternal complications shifted from women from poor households in 2005–06 to the rich households in 2015–16. In 2005–06, a higher percentage of women (45.8%) living in low SLI household had reported complications. In 2015–16, this percentage was highest among women living in high SLI households (55.7%). In 2005–06, a higher proportion of women from the Eastern region (52.1%) had complications, whereas in 2015–16, it was the women from the Central region (58.7%) that reported the highest percentage of complications.

### Service utilization factors

In 2005–06, 44.3% of women who did not receive any ANC reported more complications. But in 2015–16, 54.7% of the women who had received four or more ANC had complications. Of the women who delivered at home, 44.2% reported complications in 2005–06; in 2015–16, a higher percentage of women who delivered in institutions (54%) reported complications. In 2005–06, women who did not receive PNC reported a higher proportion of complications (44.6%). But in 2015–16, the situation had reversed with women who received PNC reporting a higher percentage of complications (54.7%) than those who did not.

### Birth outcome factors

Women who gave birth to twins had more complications in both 2005–06 and 2015–16 (52.3% and 62.9% respectively) than those who delivered one child. Women who gave birth to undersized babies reported more complications in both 2005–06 and 2015–16 (47.9% and 57.3% respectively).

Table 2 shows the results of logistic regression analysis of maternal complications in the sample in the survey periods 2005–06 and 2015–16.

**Table 2:** Odds Ratio for maternal complication over background characteristics in India, (NFHS-3 & 4, 2005–06 and 2015–16)

Variables	NFHS-3, 2005–06 (*N* = 36,850)	NFHS-4, 2015–16 (*N* = 190,898)
	**OR (95% CI)**	**OR (95% CI)**
**Age (years)**		
15–19	Ref	Ref
20–24	0.98(0.89–1.08)	0.99(0.94–1.05)
25–29	0.99(0.89–1.1)	0.99(0.93–1.05)
30–34	0.98(0.87–1.1)	0.99(0.93–1.05)
35 and Above	0.98(0.86–1.11)	0.94*(0.88–1.02)
**Education**		
No education	Ref	Ref
Primary	1.09**(1.02–1.17)	1.04**(1.01–1.07)
Secondary	0.99(0.93–1.05)	1.01(0.98–1.04)
Higher	0.96(0.87–1.07)	1.03(0.99–1.07)
**Substance Use**		
No	Ref	Ref
Yes	1.21***(1.13–1.3)	1.17***(1.13–1.21)
**Anaemia Status**		
No	Ref	Ref
Yes	1.06**(1.01–1.11)	1.01(0.99–1.03)
**Parity**		
One	Ref	Ref
Two	0.81***(0.76–0.86)	0.86***(0.84–0.88)
Three	0.83***(0.77–0.9)	0.89***(0.86–0.92)
Four and above	0.88***(0.81–0.95)	0.93***(0.89–0.96)
**BMI Status**		
Underweight	Ref	Ref
Normal	1.06**(1.01–1.12)	1.06***(1.04–1.09)
Obese	1.23***(1.13–1.35)	1.23***(1.19–1.27)
**Residence**		
Urban	Ref	Ref
Rural	1.13***(1.07–1.19)	1.06***(1.03–1.08)
**Religion**		
Hindu	Ref	Ref
Muslim	1.24***(1.16–1.32)	1.15***(1.12–1.18)
Others	1.23***(1.14–1.33)	1.30***(1.25–1.34)
**Caste**		
SC/ST	Ref	Ref
Non SC/ST	1.01(0.96–1.07)	0.98**(0.96–1.02)
**SLI**		
Low	Ref	Ref
Medium	0.93**(0.88–0.98)	0.95***(0.92–0.97)
High	0.96(0.88–1.05)	1.00(0.96–1.04)
**Region**		
North	Ref	Ref
Central	1.07*(1.03–1.15)	1.26***(1.22–1.29)
East	1.31***(1.21–1.42)	1.10***(1.06–1.13)
Northeast	1.03(0.94–1.12)	0.57***(0.55–0.60)
West	0.91**(0.84–0.99)	0.75***(0.72–0.78)
South	0.71***(0.65–0.77)	0.93***(0.90–0.97)
**Number of ANC visits**		
No Visit	Ref	Ref
1–3 Visits	0.99(0.92–1.05)	1.01(0.98–1.04)
4 & more Visits	1.07*(0.99–1.15)	1.08***(1.04–1.11)
**Place of delivery**		
Home	Ref	Ref
Institutional	1.17***(1.10–1.26)	1.05***(1.02–1.08)
**PNC**		
No or more	Ref	Ref
Within 48 h	0.95*(0.89–1.01)	1.11***(1.08–1.13)
**Mode of delivery**		
Normal	Ref	Ref
Caesarean	1.23***(1.14–1.33)	1.24***(1.20–1.27)
**Multiple births**		
Single	Ref	Ref
Twin	1.52***(1.20–1.92)	1.32***(1.19–1.45)
**Size of child**		
Large	Ref	Ref
Average	0.93**(0.88–0.99)	0.87***(0.85–0.89)
Small	1.17***(1.09–1.25)	1.07***(1.03–1.10)

*Ref* Reference Group, *OR* Odds Ratio, *C.I* Confidence interval; ***p-value < 0.01, **p-value < 0.05, *p-value < 0.10; *NFHS* National Family Health Survey, *BMI* Body Mass Index, *SLI* Standard of Living Index, *ANC* Anti-Natal Care, *SC/ST* Scheduled caste/Scheduled tribe, *PNC* Post-Natal Care, *C-Section* Caesarean Section

### Individual factors

The study did not find a significant relationship between age and education of the women with maternal complications in both survey periods. Women who were exposed to substance use were, respectively, 22% and 17% more likely to suffer from maternal complications in 2005–06 [OR = 1.22; CI: 1.13–1.30] and 2015–16 [OR = 1.17; CI: 1.13–1.21]. In addition, anemic women were 6% more likely to have maternal complications in 2005–06 than those who were not anemic [OR = 1.06; CI: 1.01–1.11]. But in 2015–16, this relationship was found to be insignificant.

One finding of interest is that women with four and higher parity had 12% and 7% lower likelihood of suffering from maternal complications than mothers who had parity one in the respective survey periods of 2005–06 [OR = 0.88; CI: 0.81–0.95] and 2015–16 [OR = 0.93; CI: 0.89–0.96]. Obese women were 23% more likely to have maternal complications than women who were underweight in both 2005–06 [OR = 1.23; CI: 1.13–1.35] and 2015–16 [OR = 1.23; CI: 1.13–1.27].

### Household factors

The results of logistic regression also show that rural women were, respectively, 13% and 6% more likely to suffer from maternal complications in 2005–06 [OR = 1.13; CI: 1.07–1.19] and 2015–16 [OR = 1.06; CI: 1.03–1.08]. Women with middle SLI were found to be less likely to report complications than women from households with lower SLI in both 2005–06 and 2015–16. In 2005–06, women from the eastern region were found to be 31% more likely to have maternal complications [OR = 1.31; CI: 1.21–1.42] in comparison with women from the northern region. In 2015–16, it was women from the central region who were 26% more likely to suffer from maternal complication [OR = 1.26; CI: 1.22–1.29] in comparison with women from the northern region.

### Service utilization factors

Women who had four or more visits of ante-natal care (ANC) had a higher likelihood of having complications by 7% in 2005–06 [OR = 1.07; CI: 0.99–1.15], and 8% in 2015–16 [OR = 1.08; CI: 1.04–1.11]. Among women who had institutional deliveries, the likelihood of having complications was higher by, respectively, 17% and 5% in 2005–06 [OR = 1.17; CI: 1.10–1.26] and 2015–16 [OR = 1.05; CI: 1.02–1.08].

In 2005–06, women who did not receive postnatal care (PNC) had 5% [OR = 0.95; CI: 0.89–1.01] lower chances of suffering from maternal complications. In 2015–16, women who received PNC were 11% more likely to have maternal complications than women who had no PNC [OR = 1.11; CI: 1.08–1.13].

### Birth outcome factors

As expected, women who delivered their babies through C-sections were, respectively, 23% and 24% more likely to have maternal complications than women who had normal childbirth in 2005–06 [OR = 1.23; CI: 1.14–1.33] and 2015–16 [OR = 1.24; CI: 1.20–1.27]. In comparison with women who delivered a single child, those who had twins were more likely to have maternal complications by 52% in 2005–06 [OR = 1.52; CI: 1.20–1.92] and 32% in [OR = 1.32; CI: 1.19–1.45].

Women who delivered average-sized children were, respectively, 7% and 13% less likely to have maternal than those who had large-sized children in 2005–06 [OR = 0.93; CI: 0.88–0.99] and 2015–16 [OR = 0.87; CI: 0.85–0.89].

**Fig. 3:**
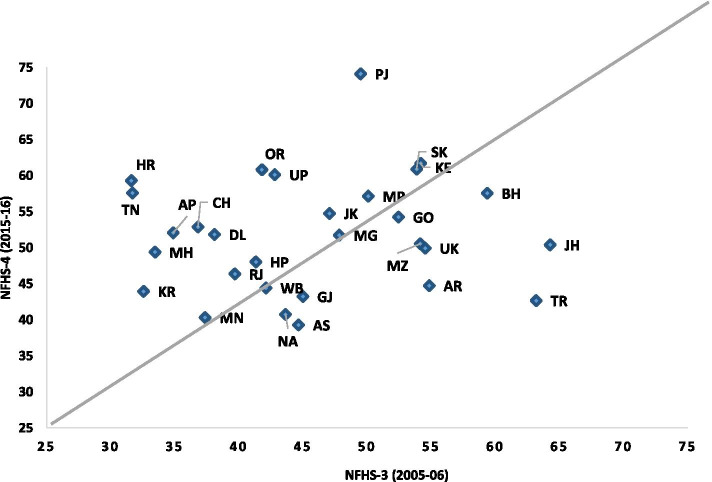
Scatter Plot for maternal complications in Indian states between 2005–06 and 2015–16

Figure 3 is a scatter plot of maternal complications in the states of India for the ten year period from 2005–06 to 2015–16. The incidences of maternal complications are seen to have increased in 20 of 29 states (above the diagonal line), while a decrease can be seen in only nine. The highest increases in the reporting of maternal complications are observed in Punjab and Haryana, followed by Tamil Nadu. On the other hand, the decrease in reported maternal complications was most in Tripura, followed by Jharkhand. In 2015–16, about 60% or more of the women in the sample from the states of Punjab, Sikkim, Kerala, Odisha, Uttar Pradesh, and Haryana reported maternal complications.

The results of decomposition analysis of the changes in maternal complications from 2005–06 to 2015–16 are presented in Table 3. As mentioned earlier also, the Fairlie decomposition method was used for analysis. This method could tell that if certain factors (or variables) had contributed to an increase or decrease in the prevalence of maternal complications during the period of study.

**Table 3:** Decomposition results for contribution of predictor variables in change of self-reported maternal complications during NFHS 2005–06 to 2015–16, India

Variables	% Contribution		
**Individual factors**			
**Age (years)**			
15–19			
20–24	-0.29	1.14	8.0
25–29	1.00		
30–34	0.45		
35 and Above	-0.03		
**Education**			
No education			
Primary	-0.08	**2.21**	
Secondary	0.78		
Higher	1.51		
**Substance Use**			
No			
Yes	-1.40	-1.40	
**Anemia Status**			
No			
Yes	-0.14	-0.14	
**Parity**			
One			
Two	-2.65	1.91	
Three	0.27		
Four and above	4.29		
**BMI Status**			
Underweight			
Normal	0.85	4.30	
Obese	3.45		
**Household factors**			**-5.4**
**Residence**			
Urban			
Rural	-0.71	-0.71	
**Religion**			
Hindu			
Muslim	0.05	0.29	
Others	0.23		
**Caste**			
SC/ST			
Non SC/ST	0.07	0.07	
**SLI**			
Low			
Medium	-3.06	-3.68	
Higher	-0.62		
**Region**			
North			
Central	-0.89	-1.41	
East	-0.08		
Northeast	0.09		
West	-0.22		
South	-0.30		
**Service utilization factors**			**19.6**
**Number of antenatal visits**			
No Visit			
1–3 Visits	0.67	0.57	
4 & More Visits	-0.10		
**Place of delivery**			
Home			
Institutional	13.05	13.05	
**PNC**			
No or more			
Within 48 h	6.01	6.01	
**Birth outcome factors**			**-1.3**
**Type of delivery**			
Normal			
Cesarean	3.54	3.54	
**Multiple births**			
Single			
Twin	0.02	0.02	
**Size of child**			
Large			
Average	-2.81	-4.88	
Small	-2.08		
**Overall Contribution**	20.89		
**Number of Total Observation**	227,647		
**N (NFHS 2005–06)**	36,850		
**N (NFHS 2015–16)**	190,797		
**Predicted value of Complication in 2005–06**	0.44		
**Predicted value of Complication in 2015–16**	0.54		
**Difference**	0.10		
**Total explained**	0.02		

*NFHS* National Family Health Survey, *BMI* Body Mass Index, *SLI* Standard of Living Index, *ANC* Anti-Natal Care, *SC/ST* Scheduled caste/Scheduled tribe, *PNC* Post-Natal Care, *C-Section* Caesarean Section

Overall, the selected variables could explain only 21% of the increase in self-reported complications. A positive contribution shows that the prevalence of maternal variable had increased between 2005–06 and 2015–16 due to a certain factor. A negative contribution means that there is a decline in the prevalence of complications. Factors related to service utilization were the primary contributors, explaining nearly 19.6% of the change in maternal complications during the period under study. This means that they had significantly contributed towards the increase in maternal complications from 2005–06 to 2015–16. Disaggregation of these factors show that place of delivery (13.0%) contributed most towards the change in maternal complications, which was followed by PNC (6.0%).

The second most important factors were individual-related (8%). In this set of factors, BMI of the women made the highest contribution (4.3%) and followed by the educational status of women (2.2%) during the period 2005–06 to 2015–16. Age of the women contributed only about (1.1%) to the change in maternal complications. Household factors (-5.4%) and birth outcome factors (-1.3%) contributed negatively —they contributed to a reduction prevalence — to the change in maternal complications during the period under study.

Among household factors, SLI was the prime contributor (-3.7%); and among factors associated with birth outcomes size of the child was the main contributor (-4.9%) to the change in maternal complications during the period from 2005–06 to 2015–16. Another factor associated with birth outcomes was type of delivery which contributed significantly (3.54%) to the the change in maternal complications during the period under study.

## Discussion

This study first analyzed the factors associated with self-reported pregnancy complications in both rounds; and then identified the significant contributors to the increase in their prevalence between the years 2005–06 and 2015–16. We found that self-reported maternal complications had increased from 43.6% in 2005–06 to 53.7% in 2015–16. Substance use, parity, BMI, residential status, place of delivery, PNC status, mode of delivery, multiple births, and size of the child were significantly associated with the self-reported maternal complications. The selected variables explained 21% of the increase in self-reported complications from 2005–06 to 2015–16.

Service utilization is seen to be the dominant contributor to the increase in self-reported increase in maternal complications, which was followed by individual factors. We could not consider factors like pre-existing diseases of the women in the sample [28], their knowledge and awareness about maternal complications which could influence self-reporting of complications. Furthermore, as these are self-reported morbidities, it is likely that the women’s perceptions changed during the decade because of which they were able to recognize signs of maternal complications.

Our study also found a shift in the prevalence of maternal complications among women in various age groups. While prevalence was high among women aged 15 – 19 years in 2005–06, it was highest among women aged 30 – 34v years in 20,015–16. This indicates the shift is from the early pregnancy-related complications to the late pregnancy-related causes. Women aged less than 18 years and more than 35 years were at a higher risk of maternal complications [7]. Another finding of our study is that the prevalence of maternal complications among women who used substances, were anemic and had higher BMIs was greater in both rounds of the surveys. This finding validates earlier studies which reported that substance use [10], anemia [14], and overweight and obesity are significantly associated with maternal complications. Interventions for improving maternal health like providing iron-folic acid supplements for lowering the risk of anemia among pregnant women [21, 27, 29], and to reduce the risk of postpartum hemorrhage and puerperal sepsis [30], need strengthening.

Prevalence of maternal complications was found to higher among first parity women than those with second or higher parity. First time pregnant women are seen to be at a higher risk of developing complications [1, 31]. Among individual factors, increased BMI and higher education levels are significantly associated with maternal complication, while decline in substance use has contributed to a reduction in maternal complications.

In both rounds of the survey, a higher proportion of women from urban areas reported complications than their rural counterparts. Although this finding is in line with previous findings [32], a few studies have found that the prevalence of maternal complications was higher in rural areas [15, 22, 33]. Women from the middle level of affluent households had lower odds of reporting complications than those who belonged to poor or rich households. Poor women are vulnerable to maternal complications because of early pregnancy, malnutrition and lack of use of services [22, 32]. On the other hand, the higher levels of self-reporting of complications by women residing in high SLI households may be attributable to their being overweight or obese, better awareness of symptoms, as well as their perceptions about maternal health.

The study found a changing association between maternal healthcare service utilization and reporting of complications during the period under study. Women who had received ANCs, delivered in the institutions, and received PNC within 48 h of delivering their babies had a lower risk of reporting complications in 2005–06. However, the reverse was observed in 2015–16. Service utilization-related factors were seen to be the primary contributors, explaining nearly 19.6% of the change in maternal complications from 2005–06 to 2015–16. The major contributing factor related to service utilization was institutional delivery which was followed by PNC.

It is also likely that women who received maternal healthcare services were more aware of the symptoms of complications than who did not receive them and therefore, their likelihood of reporting complications could have increased with service utilization. The Government of India has taken several initiatives that are focused on maternal health. These include several flagship progammes like *Janani Suraksha Yojana, Janani Shishu Suraksha Karyakram, Pradhan Mantri Surakshit Matritva Abhiyan, LaQshya* [34]. Their impact can be seen in the increase in full ANC increased from 11.6% in 2005–06 to 21% in 2015–16, institutional deliveries from 44% in 2005–06 to 78% in 2015–16, and PNC from 44% in 2005–06 to 62% in 2015–16 [24].

These programs have also reduced the inequity in maternal healthcare by covering more women from the lower socio-economic strata with these services, especially institutional deliveries. This was achieved through a cash incentive scheme, the *Janani Suraksha Yojana* [35]. The increased access to maternal healthcare services by women from the lower socioeconomic strata, who were also at a higher risk of complications due to malnutrition, early pregnancy, can explain these changes in the association of various factors with maternal complications [21].

Our study also found that C-section births contributed more to the differences in self-reported complications. Previous research stated a sharp increase (doubled) in cesarean deliveries during the last decade (2006–16). It is widely accepted that cesarean deliveries are significantly associated with maternal complications [36]. Moreover, babies born after a C-section are at higher risk of hormonal imbalances, physical abnormalities, and medical exposure [37]. Another finding from both rounds of the survey is that women who had twin births reported more complications than those delivered a single child [17]. The study found decreasing odds of complication among women with low/average birth size during the last decade (2006–16). Univariate analysis also showed that the percentage of large- and small-sized children has declined with time (Additional file 1). Therefore, in our decomposition analysis, the reduction in the prevalence of small size babies contributed significantly to the decline in self-reported maternal complications.

## Conclusions

The study found a significant increase in the prevalence of self-reported maternal complications in India between 2005–06 and 2015–16. The increase in prevalence may be partly attributed to selected individual characteristics, household’s socio-economic status, maternal service utilization, and birth outcomes. Increase in maternal BMI and education levels were found to have contributed to the increase in self-reported complications. The decline in substance use was associated with reduced maternal complications.

Target interventions for reducing maternal complications are required for women from households with lower SLI, and those from the central and eastern parts of India. Additionally, as found in this study, focused efforts are needed to increase utilization of maternal healthcare services to address the problem of maternal complications. In the decade between 2005–06 and 2015–16, there has been an impressive rise in the utilization of maternal healthcare services, especially by women from the poorer sections of society, which is largely on account of various maternal healthcare programs. These programs might increased awareness among women and facilitated the recognition of symptoms of maternal complications, thereby leading to increase in self-reporting.

Another finding of note is the reduction in the proportion of low-birth-weight babies, and that of maternal morbidity during the period under study. The high proportion of unexplained increase in maternal complications may be because of the changes in reporting behavior since women tend to recognize symptoms of complications better and earlier as their awareness and knowledge increase with time.

## Supplementary Information

**Additional file 1**. Percentage distribution of background characteristics for women in India, 2005-06 and 2015-16.

## Data Availability

NFHS-4 data can be obtained on request from the International Institute for Population Sciences, Mumbai, or the DHS. The survey tools used and complete report for India are available on the website [26].

## Abbreviations

WHO: World Health Organization
NFHS: National Family Health Survey
SC: Scheduled Caste
ST: Scheduled Tribe
OBC: Other Backward classes
SLI: Standard of living index
BMI: Body Mass Index
ANC: Antenatal check-up
PNC: Postnatal check-up
OR: Odds ratio
NRHM: National Rural Health Mission
