# Supplementary material for: An assessment of the role of socio-economic, maternal and service utilization factors in increasing self-reported maternal complications in India
Source: BMC Pregnancy Childbirth. 2021 Jul 21;21:519. doi: 10.1186/s12884-021-03997-x (PMC8296634; doi:10.1186/s12884-021-03997-x)
Supplement: Supplementary file 1 — Additional file 1. Percentage distribution of background characteristics for women in India, 2005-06 and 2015-16. [file 12884_2021_3997_MOESM1_ESM.docx]

| **Additional 1: Percentage distribution of background characteristics for women in India, 2005-06 and 2015-16** | | | | |
| --- | --- | --- | --- | --- |
| **Variables** | **NFHS 2005-06** | | **NFHS 2015-16** | |
|  | **Sample** | **Percentage** | **Sample** | **Percentage** |
| **Age (years)** |  |  |  |  |
| 15-19 | 2,158 | 5.86 | 5,899 | 3.09 |
| 20-24 | 11,268 | 30.58 | 56,183 | 29.43 |
| 25-29 | 12,435 | 33.74 | 70,169 | 36.76 |
| 30-34 | 6,993 | 18.98 | 37,331 | 19.56 |
| 35 and above | 3,996 | 10.84 | 21,316 | 11.17 |
| **Education** |  |  |  |  |
| Illiterate | 14,095 | 38.25 | 55,165 | 28.90 |
| Literate | 22755 | 61.76 | 135733 | 71.1 |
| **Substance use** |  |  |  |  |
| No | 31,461 | 85.38 | 1,71,911 | 90.05 |
| Yes | 5,389 | 14.62 | 18,987 | 9.95 |
| **Anemia status** |  |  |  |  |
| No | 14,428 | 43.26 | 84,394 | 44.97 |
| Yes | 18921 | 56.74 | 103288 | 55.03 |
| **Parity** |  |  |  |  |
| One | 10,394 | 28.21 | 61,807 | 32.38 |
| Two | 10,934 | 29.67 | 62,484 | 32.73 |
| Three | 6,297 | 17.09 | 33,064 | 17.32 |
| Four and above | 9,225 | 25.03 | 33,543 | 17.57 |
| **BMI status** |  |  |  |  |
| Underweight | 10,065 | 33.39 | 40,119 | 24.59 |
| Normal | 16,797 | 55.73 | 98,398 | 60.31 |
| Obese | 3,280 | 10.88 | 24,645 | 15.10 |
| **Residence** |  |  |  |  |
| Urban | 14,527 | 39.42 | 47,833 | 25.06 |
| Rural | 22,323 | 60.58 | 1,43,065 | 74.94 |
| **Religion** |  |  |  |  |
| Hindu | 25,806 | 70.03 | 1,38,343 | 72.47 |
| Muslim | 5,851 | 15.88 | 29,309 | 15.35 |
| Others | 5,193 | 14.09 | 23,246 | 12.18 |
| **Caste** |  |  |  |  |
| SC/ST | 12064 | 32.74 | 73059 | 38.27 |
| Non SC/ST | 24786 | 67.26 | 117839 | 61.73 |
| **SLI** |  |  |  |  |
| Low | 15,621 | 42.39 | 37,458 | 19.62 |
| Medium | 16,284 | 44.19 | 118,999 | 62.34 |
| High | 4,945 | 13.42 | 34,440 | 18.04 |
| **Region** |  |  |  |  |
| North | 6,557 | 17.79 | 36,079 | 18.9 |
| Central | 7,875 | 21.37 | 52,952 | 27.74 |
| East | 5,847 | 15.87 | 39,243 | 20.56 |
| Northeast | 6,965 | 18.90 | 28,825 | 15.10 |
| West | 4,178 | 11.34 | 13,892 | 7.28 |
| South | 5,428 | 14.73 | 19,907 | 10.43 |
| **Number of ANC visits** |  |  |  |  |
| No visit | 7,219 | 19.59 | 35,496 | 18.59 |
| 1-3 visits | 13,012 | 35.31 | 65,964 | 34.55 |
| 4 & more visits | 16,619 | 45.1 | 89,438 | 46.85 |
| **Place of delivery** |  |  |  |  |
| Home | 19,106 | 51.85 | 42,713 | 22.37 |
| Institutional | 17744 | 48.15 | 148185 | 77.63 |
| **PNC** |  |  |  |  |
| No or more | 20,549 | 55.76 | 71,233 | 37.31 |
| Within 48hrs | 16,301 | 44.24 | 1,19,665 | 62.69 |
| **Mode of delivery** |  |  |  |  |
| Normal | 32,389 | 87.89 | 1,61,160 | 84.42 |
| C-Section | 4,461 | 12.11 | 29,738 | 15.58 |
| **Multiple births** |  |  |  |  |
| Single | 36,529 | 99.13 | 1,89,143 | 99.08 |
| Twin | 321 | 0.87 | 1,755 | 0.92 |
| **Size of child** |  |  |  |  |
| Large | 8,450 | 22.93 | 33,476 | 17.55 |
| Average | 20,334 | 55.18 | 1,30,586 | 68.44 |
| Small | 8,066 | 21.89 | 26,735 | 14.01 |

*NFHS: National Family Health Survey; BMI: Body Mass Index; SLI: standard of living index; ANC: Anti-Natal Care; SC/ST: Scheduled caste/Scheduled tribe; PNC: Post-Natal Care ; C-Section:* *Caesarean Section*

^ Sample is less because BMI status has missing values not being presented in the table. (However, it was a separate category while running regression analysis so that sample reduction problem can be tackled).


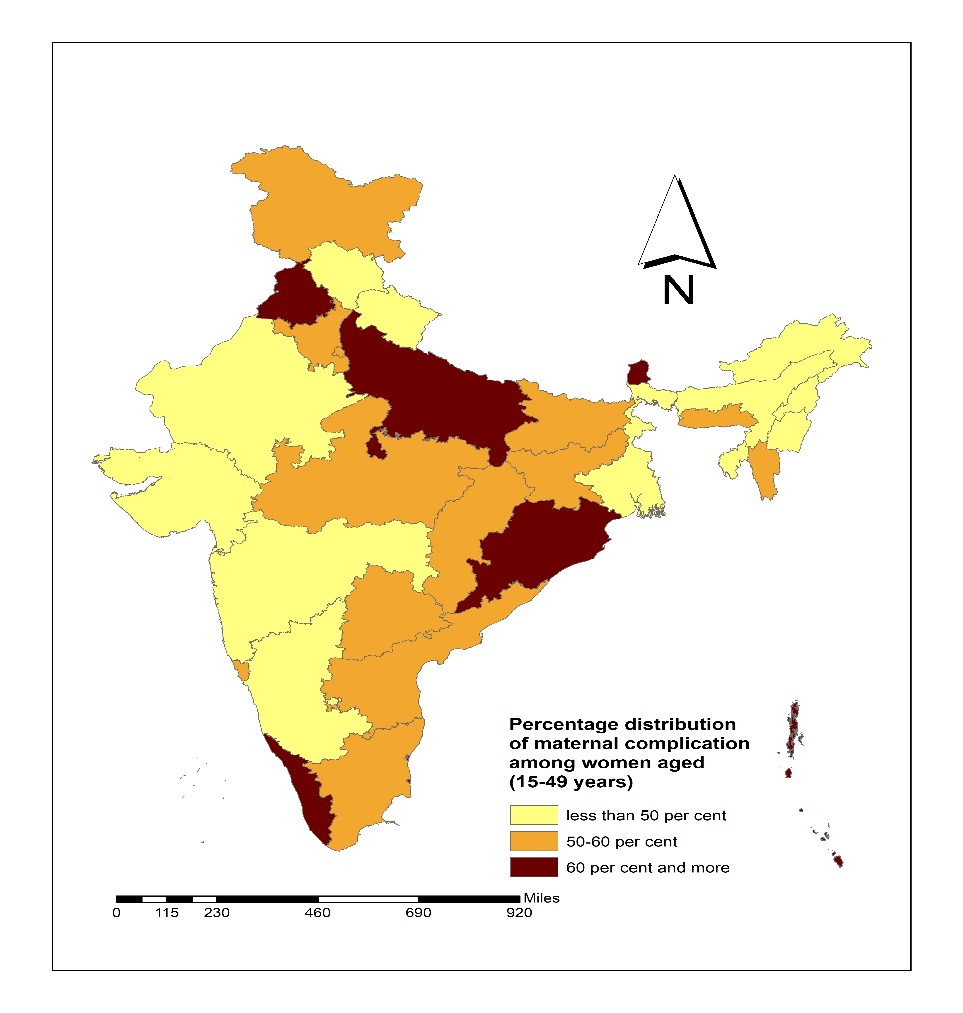

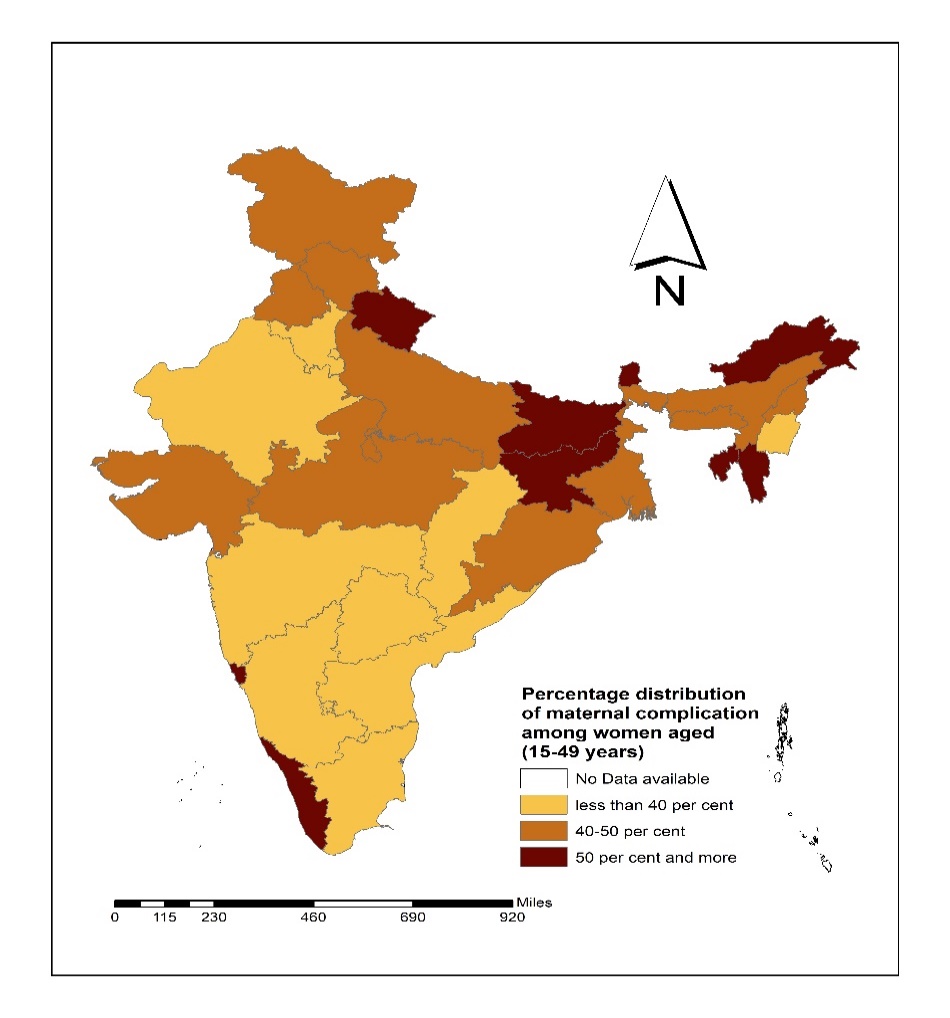
NFHS-3 MAP NFHS-4 MAP

**Source:** Authors analyzed the data and constructed maps.
